# Long-term smoking contributes to aging frailty and inflammatory response

**DOI:** 10.17305/bb.2024.11722

**Published:** 2025-01-17

**Authors:** Huijin Hou, Yidi Chai, Ting Zhang, Yue Liang, Lan Huang, Xu Cao, Shufang Liang

**Affiliations:** 1 China Tobacco Sichuan Industrial Co., Ltd., Chengdu, China; 2 Harmful Components and Tar Reduction in Cigarette Key Laboratory of Sichuan Province, Chengdu, China; 3 Department of Biotherapy, Cancer Center and State Key Laboratory of Biotherapy, West China Hospital, Sichuan University, Chengdu, China

**Keywords:** Smoking, frailty, tumor necrosis factor-α, TNF-α, interleukin-1 β, IL-1β, inflammation

## Abstract

In recent years, the health challenges linked to frailty in the elderly, particularly those worsened by cigarette smoke, have become more pronounced. However, quantitative studies examining the impact of smoking dosage on frailty in this population remain limited. To address this gap, we developed a model using smoke-exposed elderly mice. Fifteen-month-old C57BL/6J mice were exposed to smoke from two burning cigarettes for 15 min in a whole-body chamber. This exposure occurred 4, 6, and 8 times daily for 30 days, representing low, medium, and high smoking dosages, respectively. Frailty levels were assessed through rotation and grip strength tests, alongside lung histopathology and inflammatory factor protein expression analyses across the three dosage groups. Additionally, we used the Gene Expression Omnibus (GEO) database to validate the correlation between frailty and inflammation in elderly smokers, facilitating cross-comparisons between animal model findings and human sample data. Our results show that mice exposed to high-dose smoking were significantly more prone to frailty, with notable reductions in maximal grip strength (*P* < 0.01) and drop time (*P* < 0.001). Among human samples, 69.2% of elderly smokers exhibited a frailty phenotype, compared to just 15.4% of nonsmokers. Both smoking-exposed mice and elderly smokers demonstrated upregulation of tumor necrosis factor-α (TNF-α) and interleukin-1 β (IL-1β) in lung tissue and serum. Mechanistically, this upregulation activates the NF-κB signaling pathway. Our findings quantitatively link smoking-induced frailty to increased levels of TNF-α and IL-1β, providing experimental evidence for the diagnosis and prevention of frailty in elderly populations.

## Introduction

With the trend of an aging society, frailty poses a significant challenge to global health-management systems. Frailty is a disease state influenced by a combination of physiological and pathological factors that increase with age. It is primarily characterized by an organism’s heightened vulnerability and reduced ability to cope with stressors [1, 2]. Currently, frailty is gaining recognition as an emerging health threat among older adults [3, 4]. Frail individuals are at increased risk for falls, fractures, hospitalization, reduced quality of life, medical complications, and, in severe cases, life-threatening conditions due to inadequate care [5–8].

Frailty is shaped by various factors, including chronic diseases, smoking, obesity, alcohol consumption, and other unhealthy lifestyle habits. It can be screened and assessed using tools such as the frailty index (FI) model, which evaluates physical signs like weight loss, fatigue, reduced physical activity, slower walking speed, and weaker grip strength. When three or more of these characteristics are present, a person is considered frail [6]. Cigarette smoke and secondhand smoke contain harmful substances, such as nicotine, carbon monoxide, and tar. Smoking not only contributes to frailty but also accelerates its progression, as several studies have identified smoking as a significant factor influencing debilitation [1–5, 9–13]. Smoking damages a wide range of tissues and organs, making it a leading cause of diseases, such as cardiovascular disease, respiratory illnesses, and cancer [7–9]. These conditions can reduce mobility, weaken mental and physical health, and exacerbate frailty. Using the Mendelian randomization (MR) approach, researchers have found a potential causal relationship between smoking and frailty, concluding that lifetime smoking increases the risk of frailty by 46% [14]. However, few studies have examined how smoking severity correlates with frailty risk [15]. Additionally, exposure to secondhand smoke significantly increases the likelihood of physical frailty [12, 13, 16–18].

Although harmful substances in cigarettes have been shown to trigger inflammation, leading to the elevated expression of inflammatory factors [19], there remains a lack of clear quantitative evaluations regarding the dose-effect relationship between secondhand smoke exposure and physical debilitation. In this study, we aimed to establish an aged smoking mouse model to quantitatively analyze the dose-effect relationship between secondhand smoke exposure duration, inflammatory molecule expression, and physical frailty status. Inflammation is a common consequence of cigarette exposure, during which inflammatory cells are recruited to the lungs and release protein-hydrolyzing enzymes that cause tissue damage [20–22]. Chronic inflammation, in turn, leads to the accumulation of large amounts of inflammatory factors in the bloodstream, resulting in decreased muscle mass and impaired lower-extremity mobility. Additionally, chronic inflammation has been associated with depression in older adults and shares overlapping characteristics with frailty definitions. For instance, elevated inflammatory parameters are strongly linked to disability, as demonstrated in comprehensive analyses of older adults [23]. In debilitated older adults, serum levels of CRP, tumor necrosis factor-α (TNF-α), IL-6, leukocytes, and fibrinogen are significantly increased [24]. The pathogenesis of frailty is highly complex, with its symptoms partially linked to chronic inflammation and aging. Frailty itself can be classified into mild, moderate, and severe stages, which are also associated with age-related changes and smoking [25]. While several animal models of frailty—such as drug-induced pain models [26, 27]—have been developed to investigate frailty-related molecular markers and underlying mechanisms, few studies have quantitatively analyzed the dose–effect relationship between smoking and frailty [15, 16]. In our study, we evaluated the impact of smoking behavior on mouse frailty models and characterized smoking-induced frailty using the frailty phenotype (FP) criteria. Additionally, we identified and validated molecular markers based on altered inflammatory factors in conjunction with human smoking data, providing a foundational experimental basis for the diagnosis and prevention of smoking-induced frailty in humans.

## Materials and methods

### Aged mouse model

Ten-month-old male C57BL/6J mice were obtained from the Animal Care Committee of Sichuan University (Chengdu, China) and maintained under standard environmental conditions (23 ± 1 ^∘^C, 12-h dark/light cycle). All experiments involving these mice were approved by the Institutional Animal Care and Treatment Committee of Sichuan University, China (Approval No. 20220725003) and conducted in accordance with ARRIVE guidelines for animal studies. Physiological changes in mice typically begin around 10 months of age, which corresponds to approximately 40 years of age in humans [28]. The mice were housed in the West China Animal Laboratory until they reached 15 months of age (approximately 51 human years). At this age, a C57BL/6J mouse model was established. Mice were randomly assigned to weight-matched groups to eliminate baseline weight differences, and their weights were monitored daily throughout the experiments.

### Cigarette-smoke exposure

**Figure 1.**
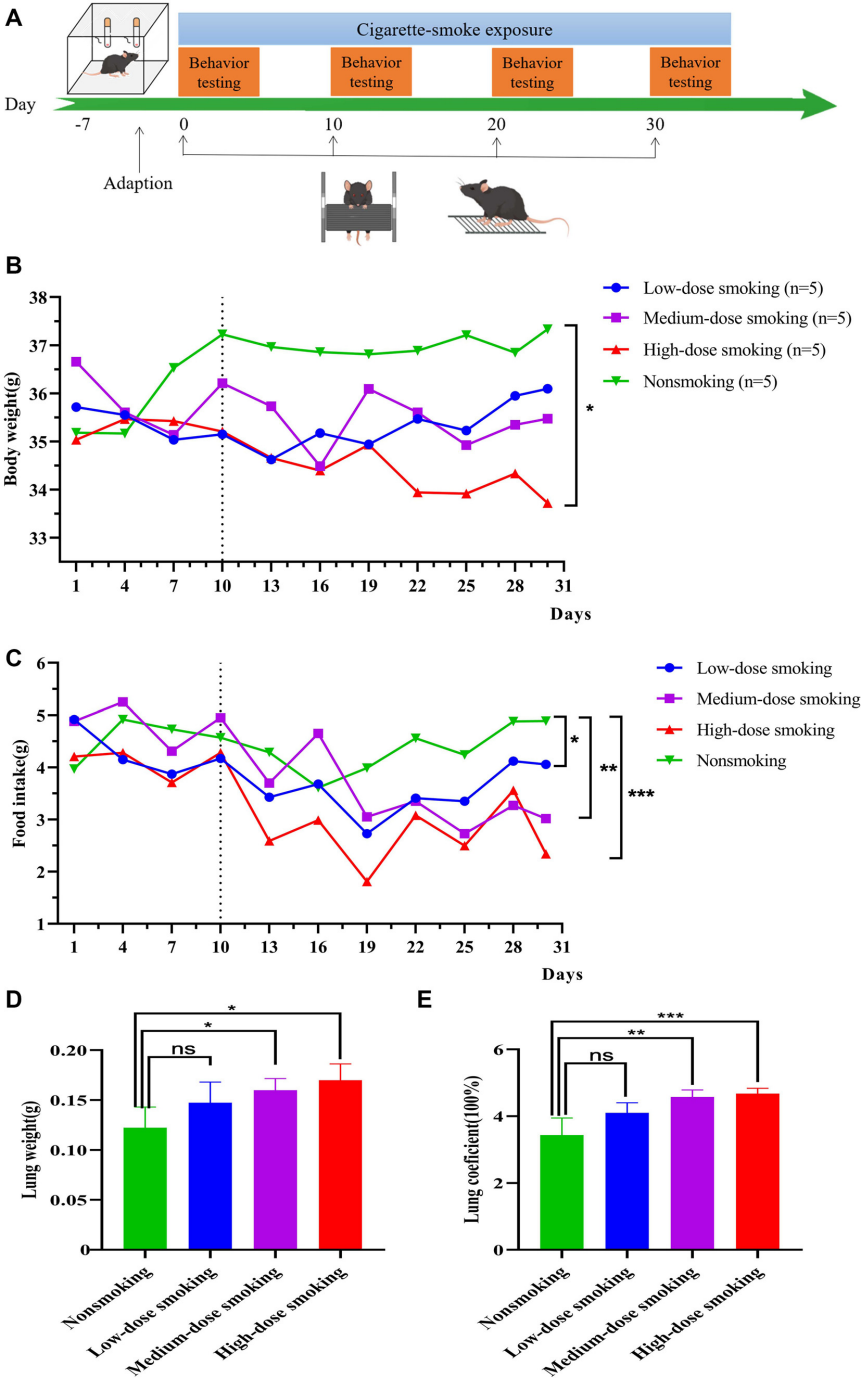
**Smoking has influence on body weight of aged mice.** (A) Experimental process of cigarette smoke-induced aged mouse model. Low-dose smoking group with 8 cigarettes/day/mouse and 88 mg tar amount/day; medium-dose smoking group with 12 cigarettes/day/mouse and 144 mg tar amount/day; and high-dose smoking group with 16 cigarettes/day and 176 mg tar amount/day for each mouse; nonsmoking mouse without cigarette smoke expose. All smoking mice were exposed to cigarette smoke for 30 days. Five mice were included in each group (*n* ═ 5); (B) During the aging modeling period, the daily body weight of a single mouse with nonsmoking, low-dose and medium-dose smoking was slightly changed, while the body weight in the high-dose smoking mice was significantly decreased (*P* < 0.05) compared with the nonsmoking group (*n* ═ 5); (C) During the aging modeling period, the daily food intake of a single mouse with nonsmoking, low-dose, medium-dose and high-dose smoking groups were changed. Compared with the nonsmoking group, the food intake in the low-dose group (*P* < 0.05), medium-dose group (*P* < 0.01) and the high-dose smoking group (*P* < 0.001) significantly decreased (*n* ═ 5); (D) Compared with the nonsmoking group, mouse lung weight was significantly increased in the medium-dose and the high-dose smoking group (*n* ═ 5) (*P* < 0.05); (E) The lung coefficient was significantly increased in medium-dose (*P* < 0.01) and high-dose smoking mice (*P* < 0.001). Detailed statistics were provided in Table 1.

Cigarettes used in our experiments were provided by the Harmful Components and Tar Reduction in Cigarette Key Laboratory of Sichuan Province. Fifteen-month-old male C57BL/6J mice housed in a whole-body exposure chamber were exposed to cigarette smoke for 15 min. The smoke was generated from two burned cigarettes (Figure 1A). After each smoking session, the mice were placed in a smoke-free environment for 5 min to prevent breathing difficulties. Cigarette exposure was administered four, six, or eight times per day, corresponding to low-, medium-, and high-dose smoking groups, respectively.

The mice were randomly assigned to four groups: a mock group with no smoke exposure, a low-dose group (8 cigarettes/day; tar amount: 88 mg/day), a medium-dose group (12 cigarettes/day; tar amount: 144 mg/day), and a high-dose group (16 cigarettes/day; tar amount: 176 mg/day). To account for individual differences and achieve statistical significance, five mice per group were used for smoke exposure, as suggested by relevant references [29, 30]. Prior to the formal experiment, we conducted two pilot studies to optimize experimental conditions, including determining the duration of a single smoking session, the maximum tolerable smoking dose, and post-exposure handling procedures. Overall, we performed three biological replicates of the experiment, utilizing a cohort of 15 mice per group. This approach balanced the need to meet data analysis requirements, such as *t*-tests and ANOVA, with ethical considerations for animal welfare. Mice in the mock group were subjected to the same confined smoking chamber environment without smoke exposure to control for housing-related confounders. During the modeling period, the experimenter, blinded to the group assignments, recorded the body weight and food intake of each mouse daily. After 30 days of smoke exposure, mice were anesthetized with isoflurane and sacrificed to collect lung tissue. Serum was extracted from peripheral blood drawn from the retro-orbital sinus. The blood was allowed to clot at room temperature for 1 h, and the supernatant was collected by centrifugation at 2000 × *g* for 15 min at 4 ^∘^C for further analysis.

### Frailty phenotype (FP)

We evaluated the mouse frailty model by referencing the frailty criteria used in humans, including tests for rotation and grip strength [9]. To assess grip strength, we performed an inverted-cling grip test using a grip strength meter to measure the maximum grasping force of the mice. During this test, each mouse was placed on a designated platform and encouraged to grip a force-sensitive sensor with its forepaws. The experimenter gently pulled the mouse’s tail until it released its grip. The peak grasping strength, recorded in Newtons (N), was displayed on the meter. Each mouse underwent three trials, and the average value was calculated to evaluate muscle strength. To validate ambulation and muscular coordination, we conducted the rotarod test using rotating equipment. Each mouse was acclimatized for 2 min at a rotational speed of 20 r/min, with their heads oriented opposite to the rod’s direction of rotation (Table 1). The total duration of the experiment was 5 min, during which the drop time and maximum distances were recorded.

**Table 1:** Frailty phenotype evaluation between smoking groups and nonsmoking group in aged mice

**Group** **characteristic **	**Nonsmoking ** **(*n* ═ 5) **	**Low-dose smoking ** **(*n* ═ 5) **	**Medium-dose smoking ** **(*n* ═ 5) **	**High-dose smoking ** **(*n* ═ 5) **
The mouse maximal grip (N), mean (SD)	1.94 ± 0.15	1.82 ± 0.20	1.79 ± 0.15	1.67 ± 0.23
The drop time (s), mean (SD)	211.92 ± 93.25	94.86 ± 80.38	86.49 ± 90.70	60.97 ± 59.42
The maximal drop distance (m), mean (SD)	6.66 ± 2.93	2.98 ± 2.53	2.72 ± 2.85	1.92 ± 1.87

N is the unit of force in Newtons, s is the unit of time in seconds, and m is the unit of distance in metres.

### Western blotting

Mouse lungs were collected for protein extraction using a lysis solution containing 1% protease inhibitors. Protein expression levels were detected through protein blotting, with a blocking solution of 50 mM Tris (pH 7.5) containing 1% (v/v) Tween-20 (1247ML100, Biofrox, China) and 5% skimmed milk powder (A600669-0250, Sangon, China) to prevent non-specific binding. The primary and secondary antibodies were diluted in 50 mM Tris (pH 7.5) containing 5% skim milk. The primary antibodies used included rabbit polyclonal antibodies against interleukin-1 β (IL-1β) (ABP52932, Abbkine, China) and TNF-α (EP65189, HUABIO, China), as well as mouse polyclonal antibodies against IL-6 (EM170414, HUABIO, China), IL-18 (EM170401, HUABIO, China), and β-Tubulin (EM0103, HUABIO, China). β-Tubulin detection served as an internal control for comparison. Protein markers with molecular weights ranging from 8 to 180 kD (X10122, X-biotech, China) and 25 to 300 kD (X10125, X-biotech, China) were employed. Protein-staining signals were visualized using a Tanon 4600 automated chemiluminescence image analysis system (Tanon, China).

### Enzyme linked immunosorbent assay (ELISA)

Candidate serum biomarkers, including IL-6, IL-1β, IL-18, and TNF-α, were verified using ELISA. Data are presented as the mean ± SD, where the standard deviation represents the square root of the mean of squared deviations from the mean. Statistical significance was assessed using unpaired two-tailed *t*-tests to compare two groups. Statistical significance was set at *P* < 0.05

### Immunohistochemistry

Immunohistochemical (IHC) analysis of lung inflammation was conducted using primary antibodies, including anti-mouse IL-18 (EM170401, HUABIO, China), anti-mouse IL-1β (ABP52932, Abbkine, China), anti-mouse IL-6 (EM170414, HUABIO, China), anti-mouse TNF-α (EP65189, HUABIO, China), and anti-rabbit p65 (ET1603-12, HUABIO, China). Mouse lung tissues were embedded in paraffin, dewaxed with xylene, and rehydrated through a series of graded ethanol solutions. Endogenous peroxidase activity was blocked using a 3% hydrogen peroxide solution, followed by antigen retrieval, which was performed by heating the tissues in an autoclave for 3 min in 10 mM sodium citrate buffer (pH 6.0). To prevent nonspecific binding, normal goat serum was used as a blocking agent for 37 min at 40 ^∘^C, after which the tissues were incubated overnight at 4 ^∘^C with the primary antibody. The slides were subsequently incubated with a horseradish peroxidase (HRP)-conjugated secondary antibody for 40 min at 37 ^∘^C, followed by streptavidin–biotin complex incubation for another 40 min at the same temperature. HRP activity was visualized using a diaminobenzidine peroxide solution, and the nuclei were counterstained with hematoxylin (Bison Biotech Institute, Shanghai, China). ImageJ software was employed to statistically analyze the proportion of positively stained areas in the IHC results.

### Lung histopathologic examination and Smith score assay

The lower lobe of the mouse lung tissue was fixed in 4% paraformaldehyde, embedded in paraffin, and stained with hematoxylin and eosin (H&E). The extent of lung injury was assessed using the Smith score assay [31]. Individual sub-tissues, including alveolar and interstitial inflammation, edema, alveolar and interstitial hemorrhage, necrosis, atelectasis, and hyaline membrane formation, were scored on a scale from 0 to 4. The scoring criteria were defined as follows: 0, no injury; 1, injury present in 25% of the field; 2, injury in 50% of the field; 3, injury in 75% of the field; and 4, injury across the entire field.

### Bioinformatics analysis of persons based on GEO clinical transcription database

The GEO database (http://www.ncbi.nlm.nih.gov/geo) is a publicly accessible repository for gene transcriptional expression profiles. Transcriptome data from individuals with a smoking history and nonsmokers were curated from multiple GEO datasets for bioinformatics analysis. Three gene expression profiledatasets—GSE12585, GSE18723, and GSE37768—were selected for this study. GSE12585: This dataset contains peripheral blood mononuclear cell (PBMC) samples from 10 nonsmokers and 13 light smokers, obtained using the GPL96 platform (Affymetrix Human Genome U133A Array). GSE18723: Comprising PBMC samples from 39 smokers and 40 nonsmokers, this dataset was also generated using the GPL96 platform. GSE37768: This dataset includes lung tissue samples from 11 smokers and 9 nonsmokers, produced using the GPL570 platform (Affymetrix Human Genome U133 Plus 2.0 Array). Bioinformatics analyses were performed using R version 3.6.1 (https://cran.r-project.org/) and associated software packages from Bioconductor (http://www.bioconductor.org/). Gene expression matrices from the target datasets and probe data from the corresponding platform TXT files were preprocessed with background correction, normalization, and summarization. Differentially expressed genes (DEGs) were identified and analyzed using the Kyoto Encyclopedia of Genes and Genomes (KEGG) database.

### Human frailty subjects

Frailty subjects were recruited after obtaining informed consent and approval from the Committee on Medical Ethics of the State Key Laboratory of Biotherapy at West China Hospital, Sichuan University (2013040). This study adhered to the principles outlined in the Declaration of Helsinki, and written informed consent was obtained from all participants. Participants included patients and healthy donors. Frailty subjects were selected based on the following criteria: individuals aged 60 years or older who could walk independently or with the assistance of aids to maintain their original lifestyle. To ensure the robustness of group comparisons, subjects were matched with minimal age and gender differences between smokers and nonsmokers, ensuring sufficient sample size for data significance analysis. The final sample included 13 smokers (seven males, six females) with an average age of 72.3 ± 3.9 years, and 13 nonsmokers (nine males, four females) with an average age of 70.7 ± 5.9 years. Participants with major diseases, terminal cancer, or conditions that might interfere with the study were excluded. Additionally, subjects were instructed to refrain from vigorous activities for three days before the experiment while maintaining their usual lifestyle. During the study, participants were assessed on their physical performance, which was recorded as the number of stairs climbed and the walking distance covered without assistance. Frailty was defined based on the FI scale, with the presence of three or more of the following five parameters: Frequently feeling tired in the past four weeks; difficulty climbing 10 stairs without the assistance of mobility aids; difficulty walking 500–600 meters without the assistance of mobility aids; unintentional weight loss of up to 5% of body weight in the past month; the presence of five or more chronic diseases. The total FI score ranged from 0 to 5, with one point awarded for each criterion met. A score of three or higher classified the individual as being in a frailty state.

### Ethical statement

This study was approved by the Committee on Medical Ethics of the State Key Laboratory of Biotherapy, West China Hospital, Sichuan University (2013040). It was conducted in accordance with the Declaration of Helsinki, and informed consent was obtained from all participants. According to the standard operating procedures of the WHO Research Ethics Review Committee, a research proposal may be exempt from committee review when the data (including healthcare records and specimens) being studied already exist, are publicly available, or are recorded in a manner that ensures the investigator or research team cannot identify the individuals. GEO is a public database, and all patients included in it have already received ethical approval. Researchers can freely download relevant data from GEO to conduct research and publish articles. Our study was based on open-source data and did not disclose any personal information; therefore, there were no ethical concerns or conflicts of interest. Additionally, all experiments involving C57BL/6J mice were approved by the Institutional Animal Care and Treatment Committee of Sichuan University, China (20220725003). All procedures adhered to the applicable rules and regulations outlined in the ARRIVE guidelines.

### Statistical analysis

GraphPad Prism 8.0 and open-source R software (version 3.5.2) were used to perform statistical analyses. All data are presented as mean ± SD. Prior to significance testing, Normality and Lognormality tests were conducted to assess the normality of the data. Parametric tests were performed only when the data satisfied the criteria for normal distribution. Differences between two groups were considered significant based on *t*-tests if the *P* values were below 0.05, with the significance levels denoted as *P < 0.05; **P < 0.01; ***P < 0.001; ****P < 0.0001. For comparisons involving multiple groups, GraphPad’s one-way ANOVA was employed, followed by post hoc tests, to analyze data from four groups: nonsmoking, low-, medium-, and high-dose smoking groups.

## Results

### Decrease of body weight in smoking aged mice

Fifteen-month-old mice were exposed to cigarette smoke for 15 min at intervals of 5 min each, and the low-, medium-, and high-dose smoking mice smoked for 4, 6 and 8 times per day. The total smoking exposure lasted for one month (Figure 1A). During the former 10 experimental days, the weight and daily food intake of a single mouse normally fluctuated within a gentle range in both the nonsmoking and smoking groups. However, on day 11, the body weight and daily food intake of each mouse significantly decreased in the three smoking groups (Table 1). After a month of smoke exposure, the high-dose smoking mice showed a significant reduction in daily body weight compared to the nonsmoking group (*P* < 0.05), whereas there was no significant difference between the middle- and low-dose groups (Figure 1B).

In addition, the food intake of each mouse in the low-(*P* < 0.05) and medium-dose (*P* < 0.01) smoking groups were significantly lower than those in the nonsmoking group. Notably, high-dose smoking mice had the most significant reduction in food intake (*P* < 0.001) (Figure 1C). In general, cigarette smoking at a high dose has an obvious influence on body weight and food intake of elderly mice. In particular, cigarette smoke inhalation with a tar dose of more than 176 mg per day for a month significantly leads to an obvious decrease in mouse body weight and food intake.

Moreover, we compared the lung tissue weight and lung weight coefficient of smoking mice with those of nonsmoking mice. The lung weight coefficient was defined as the ratio of the lung weight to the body weight. Compared to the nonsmoking mice, the lung weight in the medium- and high-dose smoking mice was significantly increased (*P* < 0.05). However, it was not significantly altered in the low-dose smoking group (Figure 1D). The lung weight coefficient was 3.44% higher in the smoking group than in the nonsmoking group. The lung weight coefficients averaged 4.10%, 4.58%, and 4.68% in the low-dose, medium-dose (*P* < 0.01), and high-dose smoking groups, respectively (*P* < 0.001) (Figure 1E).

### High-dose smoking mice are prone to debilitation

**Figure 2.**
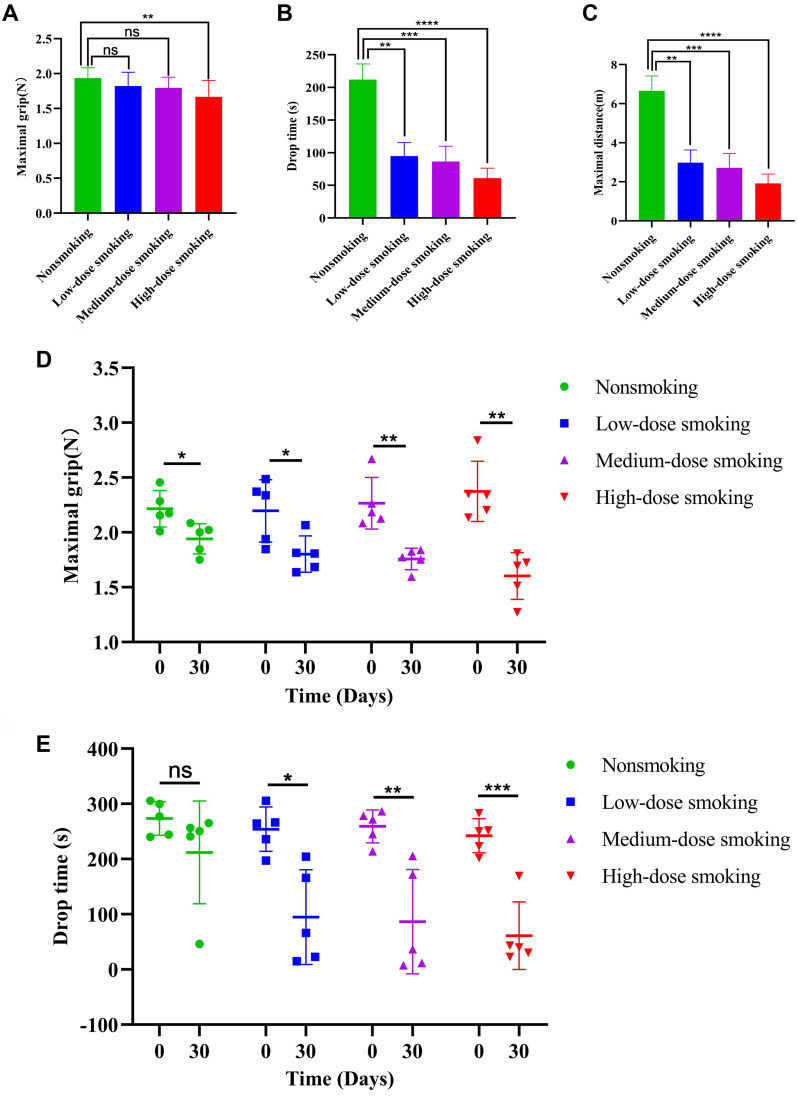
**The frailty phenotype evaluation of smoking mice by endurance assay (rotarod plus grip strength).** (A and C) The maximal grip was measured in the grip strength test, the rotarod test included the drop time and the maximal drop distance. For each group, five mice (*n* ═ 5) were included and tested on the 30th day of smoke exposure. Each mouse underwent three separate measurements, and the average of these three readings was taken as the final measurement for that mouse. (A) Compared with the nonsmoking group, the maximal grip in the high-dose group was significantly decreased (*P* < 0.01), but it was not significantly changed in the low-dose and medium-dose smoking group; (B and C) The drop time (B) and the maximal drop distance (C) was both significantly decreased in the low-dose (*P* < 0.01), medium-dose (*P* < 0.001) and high-dose smoking group (*P* < 0.0001) compared with the nonsmoking group; (D and E) We further compared within-group differences of grip strength and drop time from three group of mice before and after smoking (*n* ═ 5); (D) The grip strength of elderly low-dose, medium-dose and high-dose smoking mice decreased by 17.97% (*P* < 0.05), 22.42% (*P* < 0.01) and 32.49% (*P* < 0.01) respectively compared to before smoke exposure, while the grip strength of nonsmoking elderly mice (*P* < 0.05) only decreased by 12.38% (*n* ═ 5); (E) The elderly mice in the low-dose, medium-dose and high-dose smoking group could keep on the rotating instrument for 159.14 s (*P* < 0.001), 172.73 s (*P* < 0.01), and 181.12 s (*P* < 0.05) less than each group before smoke exposure. However, the time of the nonsmoking elderly mice was only 61.55 s less than before smoke exposure (*n* ═ 5). N: Newton.

**Figure S1.**
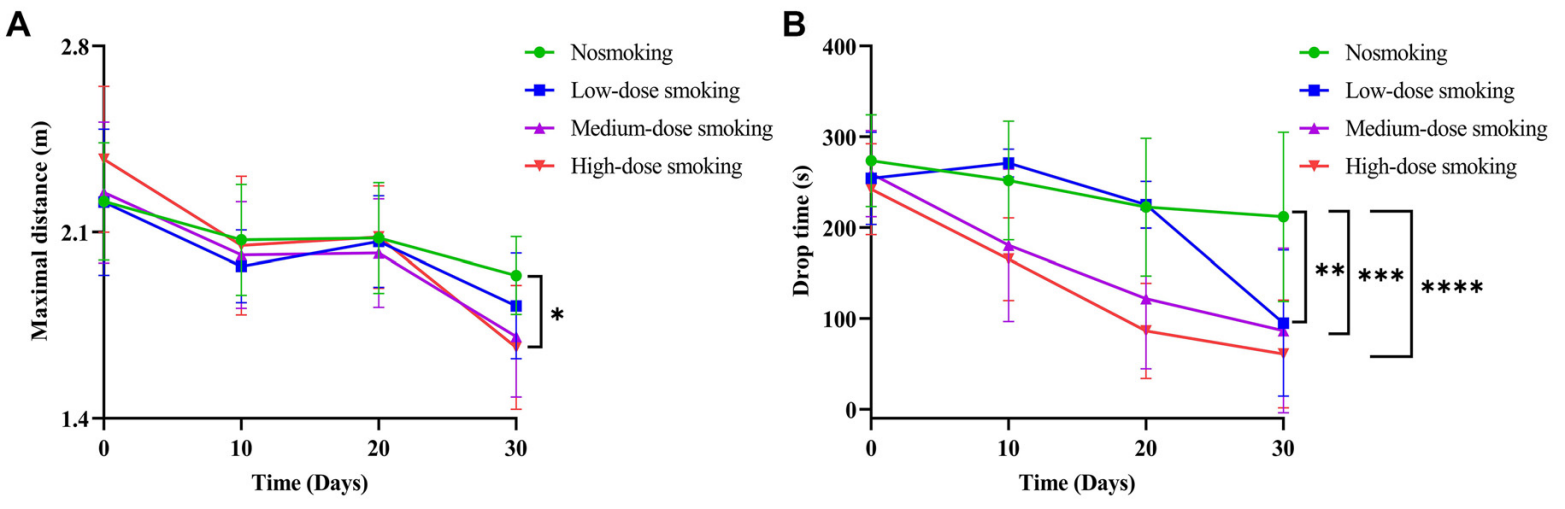
**Continuous high-dose smoking accelerates frailty in mice.** (A) The maximal grip was measured in the grip strength test. The maximum grip strength of mice was measured on the 0, 10, 20, and 30 days after smoke exposure and (B) The rotarod test included the drop time and the maximal drop distance. The drop time of mice was measured on the 0, 10, 20, and 30 days after smoke exposure. All data were shown as mean ± SD. *P < 0.05; **P < 0.01; ***P < 0.0005; ****P < 0.0001.

The FPs of elderly smoking mice were evaluated at monthly time points during smoke exposure using endurance assays, including grip strength and rotation fatigue tests. Maximal grip strength was assessed via the grip strength test, while the rotarod test measured parameters, such as drop time and maximal drop distance on the rotating equipment. At each monthly time point, three indicators were tested in both smoking and nonsmoking groups (Table 1). Compared to nonsmoking mice, the maximal grip strength of high-dose smoking mice significantly decreased (*P* < 0.01) (Figure 2A). However, no significant changes were observed in the low- and medium-dose smoking groups. Regarding drop time, a significant decrease was observed in the low-dose (*P* < 0.01), medium-dose (*P* < 0.001), and high-dose smoking mice (*P* < 0.0001) compared to the nonsmoking group (Figure 2B). Similarly, maximal drop distance was significantly reduced in all smoking groups (low-, medium-, and high-dose) (Figure 2C). These findings indicate that the endurance of elderly smoking mice is weaker than that of elderly nonsmoking mice. Further within-group comparisons were conducted to evaluate changes in the three test indicators before and after 30 days of smoke exposure. In the grip strength test, all smoking groups (low-, medium-, and high-dose) showed a continuous decline in grip strength after smoke exposure. Specifically, grip strength decreased by 17.97% (*P* < 0.05), 22.42% (*P* < 0.01), and 32.49% (*P* < 0.01). Meanwhile, the grip strength of nonsmoking aged mice also decreased by 12.38% (*P* < 0.05) over 30 days. Overall, the grip strength of high-dose smoking mice was significantly lower than that of the nonsmoking group (Figure 2D, Figure S1A).

In the rotational fatigue test, we observed a significant decrease in the duration of time spent on the rotating instrument by aged mice across all smoking groups (low-, medium-, and high-dose). Following smoke exposure, mice in these groups demonstrated reductions in persistence time of 159.14 s (*P* < 0.001), 172.73 s (*P* < 0.01), and 181.12 s (*P* < 0.05), respectively. In contrast, nonsmoking elderly mice experienced only a 61.55-s reduction in persistence time, which was not statistically significant. After 30 days of smoke exposure, all smoking groups showed a significant decrease in adherence duration on the rotating device compared to the nonsmoking group (Figure 2E and Figure S1B). Overall, aged mice exposed to cigarette smoke for prolonged periods exhibited weaker grip strength in the grip test. In the rotational fatigue test, fall time measured with the rotation meter was inversely proportional to the level of smoke exposure. Mice exposed to high doses of smoke experienced the longest cumulative smoke exposure time and fell off the device the fastest. These findings indicate that smoking contributes to the FP in elderly mice, characterized by reduced grip strength, impaired motor coordination, and decreased endurance. The degree of frailty and the prominence of these phenotypic changes were directly proportional to the amount of cigarette smoke exposure.

### IL-1β and TNF-α levels are elevated in smoke-induced debilitated mice

**Figure 3.**
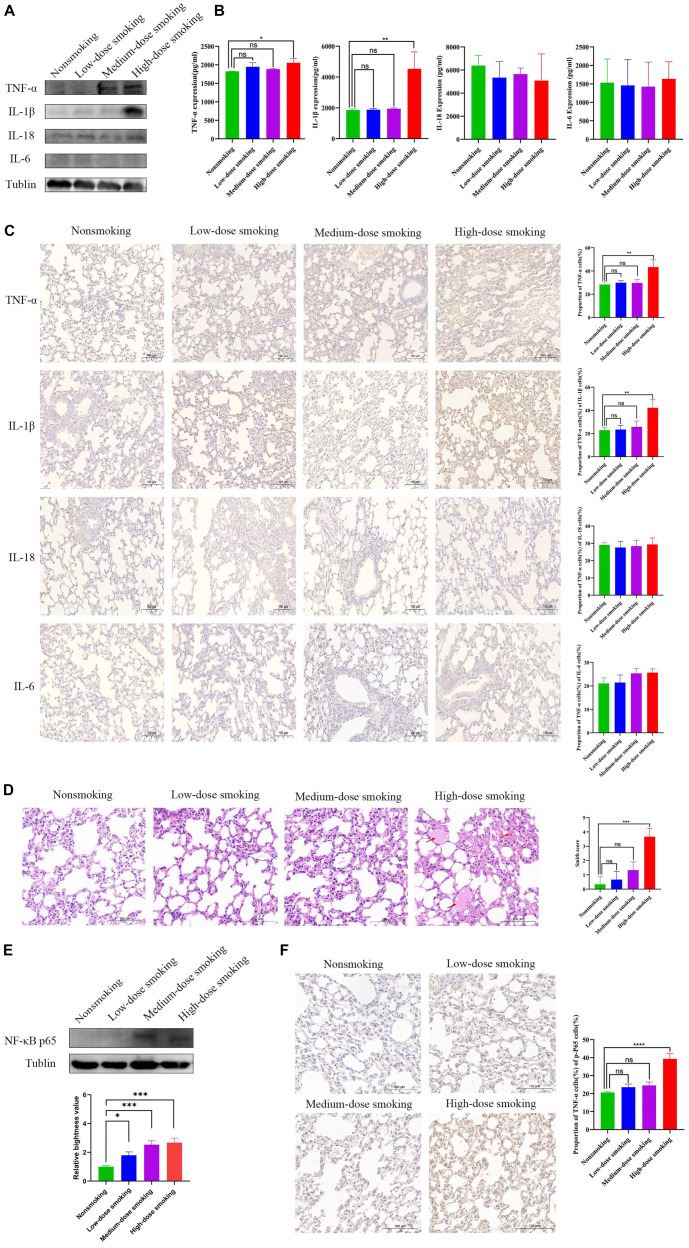
**The protein expression level of TNF-α and IL-1β is elevated in smoking-induced debilitated mice.** (A) The expression of TNF-α and IL-1β in mouse lung tissues were significantly increased in the high-dose smoking group compared with the nonsmoking group. (B) ELISA of mouse peripheral blood showed the expression of TNF-α (*P* < 0.05) and IL-1β (*P* < 0.01) was increased in the high-dose smoking group, but there was no significantly changed of serum IL-6 and IL-18 compared with the nonsmoking group. (C) IHC analysis for mouse lungs of TNF-α, IL-1β, IL-18, and IL-6 in nonsmoking group, low-dose, medium-dose and high-dose smoking group. Scale bar represents 100 µm. And the statistical results of the percentage of positive cells were presented as histograms. IHC images represented the protein expression of all mice within each group. (D) The H&E staining showed that there were obvious pulmonary edema and inflammatory infiltration in the lung tissue of mice with high-dose smoking, which were indicated by arrows. Scale bar represents 100 µm. The statistical results of the Smith score were represented as histograms. H&E pictures shown were representative of the behavior of all mice within each group. (E) Western blot showed that the expression of NF-κB p65 in mouse lung tissues was significantly increased in the high-dose smoking group compared with the nonsmoking group. And the statistical results of the relative brightness value were presented as histograms. Western blot images represent the protein expression profiles of all mice within each group. (F) Mouse lung IHC profiling of NF-κB p65 level in low-dose, medium-dose and high-dose smoking group compared with nonsmoking group. Scale bar represents 100 µm. The statistical results of the percentage of positive cells were presented as histograms. IHC images represented the protein expression of all mice within each group. IHC: Immunohistochemical; TNF-α: Tumor necrosis factor- α; IL-1β: Interleukin-1β; ELISA: Enzyme linked immunosorbent assay; H&E: Hematoxylin and eosin.

As is well known, several inflammatory markers, including TNF-α, IL-1β, IL-6, IL-18, and NF-κB, play crucial roles in the development of frailty. TNF-α, IL-1β, IL-6, and IL-18 amplify the inflammatory response by upregulating cytokine expression and modulating immune cell proliferation and differentiation, which profoundly affects immune system function [32]. Additionally, these cytokines accelerate cellular senescence and apoptosis [33, 34]. Notably, TNF-α and IL-6 are directly involved in skeletal muscle degradation. Aberrant expression of these markers can lead to tissue damage, dysfunction, and overall health decline, thus promoting the onset and progression of frailty [35]. Moreover, TNF-α and IL-1β activate the NF-κB pathway, further exacerbating frailty by inducing the expression of multiple inflammatory cytokines. Given the strong association and critical role of these inflammatory molecules in frailty, we focused on these proteins to validate their use as detection indicators in smoke-induced mice. The protein expression levels of frailty-related inflammatory factors were examined in the lung tissues of smoke-exposed elderly mice. TNF-α and IL-1β expression levels were significantly increased in the high-dose smoking group (Figure 3A). However, their expression did not significantly change in the low- or medium-dose groups. IL-18 was expressed in both smoking and nonsmoking groups, but no significant differences were observed between the two. Compared to nonsmoking mice, IL-6 and IL-18 levels showed no significant changes in the smoking groups. Additionally, the ELISA results from mouse peripheral blood were consistent with the protein expression levels in lung tissue. Serum TNF-α and IL-1β levels were significantly elevated in the high-dose smoking group, while IL-6 and IL-18 levels exhibited no notable differences compared to the nonsmoking group (Figure 3B).

In addition, inflammatory factor expression in mouse lungs was evaluated using IHC, which revealed an increase in TNF-α and IL-1β levels in the high-dose smoking groups compared to the nonsmoking group. However, no significant changes were observed in IL-18 and IL-6 levels between smoking and nonsmoking mice (Figure 3C). Based on the Smith scoring system, substantial inflammatory cell infiltration was identified in the high-dose smoking group (Figure 3D). These findings indicate that high-dose smoking induces elevated TNF-α and IL-1β expression in debilitated mice.

### TNF-α induces p65 upregulation in high-dose smoking mouse lung tissue

Based on TNF-α as the primary molecular marker linking smoking to frailty [29, 36], we further examined its involvement in the NF-κB inflammatory signaling pathway. Our investigation revealed that the expression levels of NF-κB p65 protein were significantly elevated in all three smoking groups compared to the nonsmoking group, with the most pronounced increases observed in the middle- and high-dose smoking groups (*P* < 0.001) (Figure 3E). Additionally, IHC analysis of mouse lung tissue across various smoking doses showed that p65 expression was highest in the high-dose smoking group compared to the nonsmoking, low-dose, and middle-dose groups (Figure 3F). These findings indicate that NF-κB pathway-induced inflammation plays a significant role in the development of the FP in smoking mice and is strongly correlated with smoking dose. Notably, the inflammatory phenotype emerged predominantly in aged mice exposed to high-dose smoke inhalation, which aligns with their increased susceptibility to frailty. In contrast, low-dose smoking mice exhibited minimal lung inflammation, likely because their smoking exposure did not reach the effective threshold (144 mg/day).

### Human sample database analysis indicates a strong association of smoking with inflammatory pathways

**Figure 4.**
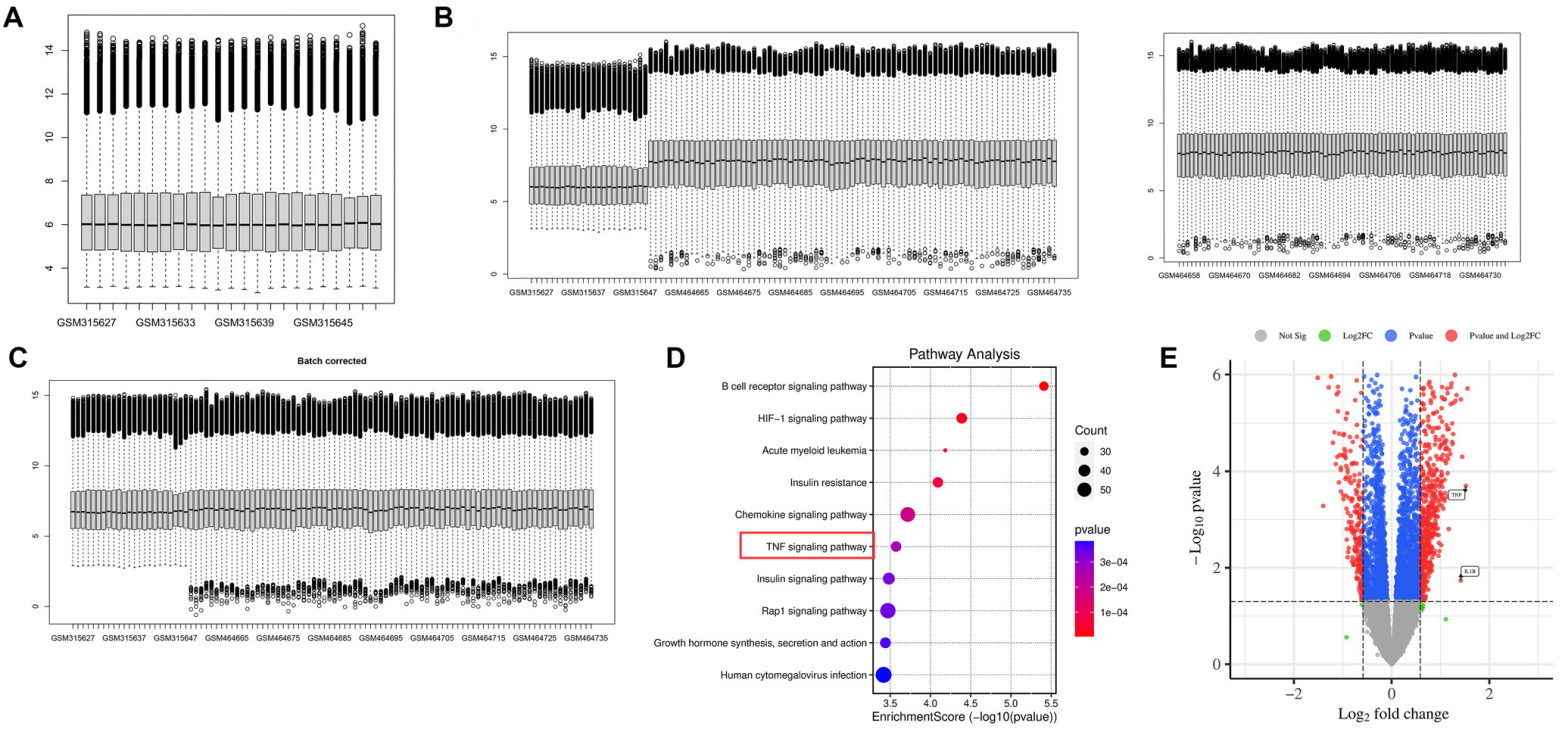
**Bioinformatics analysis of human smoking-related** **DEGs.** (A) The box plot of GSE12585 and GSE18723; (B) The two datasets before de-batching effect treatment; (C) The two datasets after de-batching effect treatment; (D) KEGG analysis of all differential genes between smokers and nonsmokers groups; (E) The volcano plot of the DEGs between smokers and nonsmokers were identified according to the conditions of a fold change FC >1.5 or FC <0.67 and an adjusted *P* value < 0.05. *TNF* and *IL-1β* showed significant upregulation. DEG: Differentially expressed gene; KEGG: Kyoto Encyclopedia of Genes and Genomes.

We selected human smoking-related datasets from the GEO platform to validate the effects of smoking in an aged mouse model. Specifically, we used peripheral blood samples from GSE12585 and GSE18723, as well as lung tissue samples from GSE37768. To ensure comparability between datasets, we performed a de-batching treatment on the two peripheral blood datasets. The gene chip data used for differential gene analysis was normalized to standardize gene expression levels across samples. A box plot was employed to visualize and correct the batch effect (Figure 4A).

Our analysis revealed that the gene expression levels in GSE12585 and GSE18723 were initially not on the same baseline, reflecting significant differences in their clinical samples and sequencing platforms (Figure 4B). Consequently, a simple dataset merge failed to provide accurate representations of true gene expression levels. However, after the de-batch effect treatment, the expression levels were aligned, confirming that the batch effect had been effectively corrected (Figure 4C). Dataset analysis identified significant differences in multiple KEGG molecular pathways associated with inflammation, most notably the TNF signaling pathway, which exhibited pronounced changes (Figure 4D). DEGs between smokers and nonsmokers were identified based on the criteria of fold change (FC > 1.5 or FC < 0.67) and an adjusted *P* value <0.05. Additionally, we observed a significant increase in TNF and IL-1β levels (Figure 4E).

### Validation of several biomarkers in human smoker’s samples

**Figure 5.**
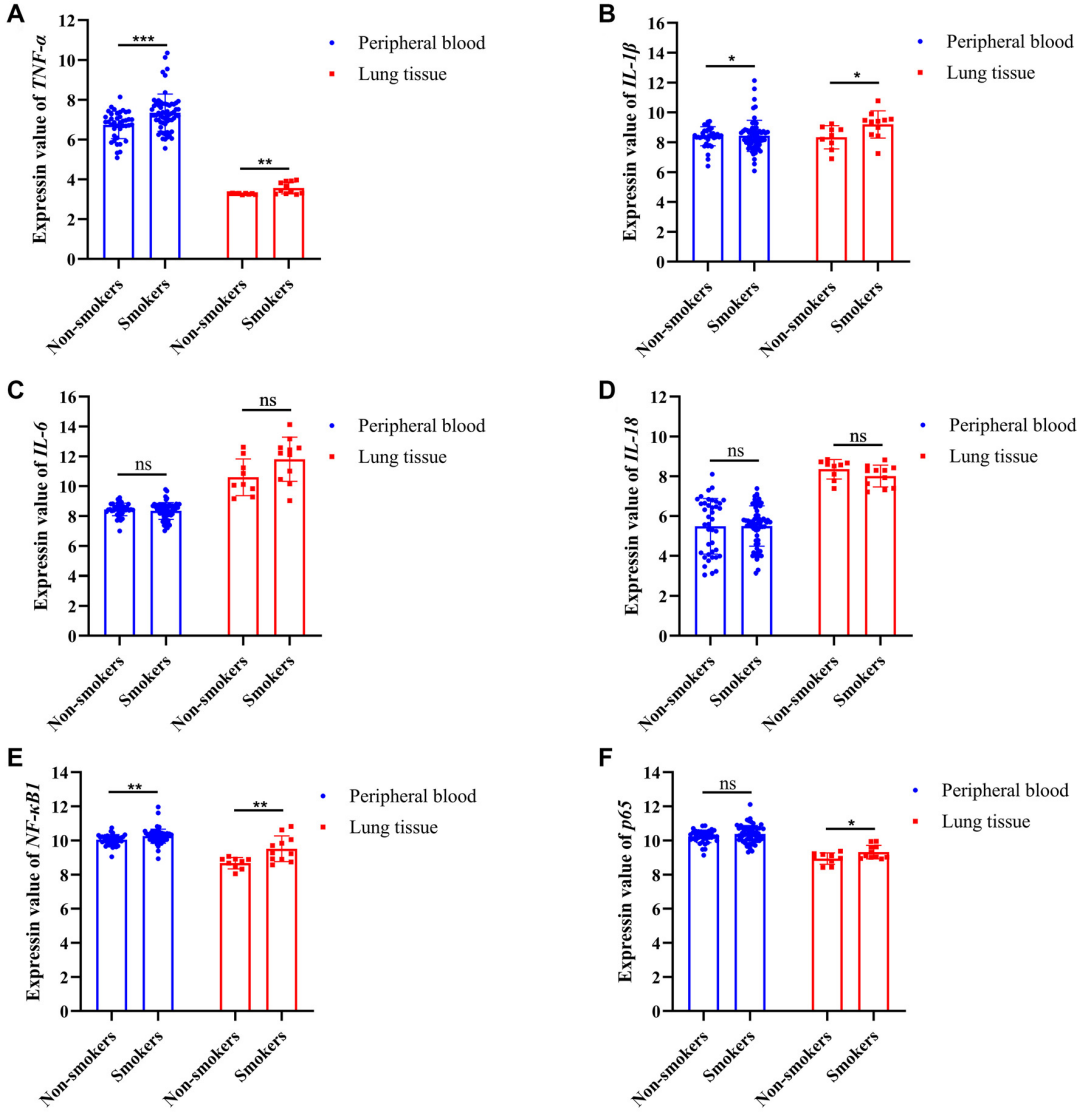
**Validation of biomarkers in human smoker’s samples.** (A–D) The gene expression level of inflammation factors in human smoker’s peripheral blood and lung tissue from the GEO database. In the peripheral blood, the expression of *TNF-α* (*P* < 0.001) and *IL-1β* (*P* < 0.05) in the smokers (*n* ═ 62) was significantly increased, but the expression of *IL-6* and *IL-18* in the smokers (*n* ═ 62) was no significantly altered compared to the nonsmokers (*n* ═ 40). Similarly, in the lung tissue, the expression of *TNF-α* (*P* < 0.01) and *IL-1β* (*P* < 0.05) in the smokers (*n* ═ 11) was significantly increased, but the expression of *IL-6* and *IL-18* in the smokers (*n* ═ 11) was no significantly altered compared to the nonsmokers (*n* ═ 9). (E and F) The gene expression level of NF-κB pathway in human smoker’s peripheral blood and lung tissue from the GEO database. In the peripheral blood, the expression of *NF-κB1* (*P* < 0.01) in the smokers (*n* ═ 62) was significantly increased, but the expression of *p65* in the smokers (*n* ═ 62) was no significantly altered compared to the nonsmokers (*n* ═ 40). While in the lung tissue, compared with the nonsmokers (*n* ═ 9), the expression of *NF-κB1* (*P* < 0.01) and *p65* (*P* < 0.05) in the smokers (*n* ═ 11) was significantly increased. TNF-α: Tumor necrosis factor-α; IL-1β: Interleukin-1β; GEO: Gene Expression Omnibus.

We further analyzed the expression of several mouse inflammatory molecules, including *TNF-α*, *IL-1β*, *IL-6*, and *IL-18*, using data from human smokers in peripheral blood (GSE12585 and GSE18723) and lung tissues (GSE37768). In both peripheral blood and lung tissue, the expression levels of *IL-6* and *IL-18* were not significantly different between smokers and nonsmokers (Figure 5A and 5B). However, the differential expression of *TNF-α* and *IL-1β* in the peripheral blood and lung tissues of smokers was evident (Figure 5C and 5D).

Additionally, we investigated the expression of key molecules in the NF-κB pathway, including *NF-κB1* and *p65*. The results revealed that both *NF-κB1* and *p65* levels were significantly elevated in smokers (Figure 5E and 5F). The analysis of human smoker datasets aligned with results from mouse simulation experiments, suggesting that the mouse smoking model accurately mirrors the sequence of biochemical reactions observed in humans after smoking. These findings further highlight that the widespread smoking-induced upregulation of inflammatory cytokines (*TNF-α* and *IL-1β*), coupled with activation of the NF-κB pathway, is a common occurrence that may promote frailty. We also explored the relationship between smoking and frailty in the older population. Clinical case data for all participants included age, sex, frailty scores, and the presence or absence of lung disease (Table 2). The mean age of older adults with smoking-induced lung disease was 72 years, compared to 70 years for those without a smoking history. Among older adults with lung disease caused by smoking, 69.2% had frailty, and 100% had a tendency toward frailty. In contrast, among older adults with no history of smoking, only 15.4% experienced frailty, and 38.5% had a tendency to develop frailty. These findings suggest that, in older adults, the occurrence of frailty is strongly influenced by smoking. Older adults with smoking-induced lung diseases are significantly more likely to experience frailty or have a higher risk of developing frailty (Table 2).

**Table 2:** The clinical data analysis between smoking and frailty in older population

**Group characteristic**	**Smoking**	**Nonsmoking**
Age (years), mean (SD)	72.3 ± 3.9	70.7 ± 5.9
Number (*N*)	13	13
Sex, *N* (Male/Female)	7/6	9/4
Frailty score ≥ 3, *N* (%)	9 (69.2%)	2 (15.4%)
Frailty score ═ 0, *N* (%)	0 (0.0)	8 (61.5%)
Frailty tendency (%)	100	38.5

Frailty tendency is defined as the proportion of frailty score greater than zero.

## Discussion

With the global trend of an aging population, frailty has emerged as a significant challenge for health-management systems worldwide. Frail older adults are at increased risk of falls, fractures, hospitalizations, diminished quality of life, medical complications, and even more serious outcomes due to inadequate care [6–8]. Frailty is clinically correlated with aging and is often assessed through observable changes in a patient’s behavior, as well as physiological changes in multiple organs [37–39]. When exposed to acute stressors, such as sudden illnesses, the weakened organs of frail individuals exhibit a reduced capacity to cope. Due to the complex pathogenic mechanisms underlying frailty, current clinical practices rely on frailty assessment tools, including the FP and the FI based on cumulative deficits [9, 40–42]. These assessments focus on indicators, such as weight loss, fatigue, reduced physical activity, slower walking speed, and weaker grip strength. A diagnosis of frailty is typically made when a patient meets three or more of these criteria. However, these tools do not incorporate pathological biomarkers to confirm frailty. The onset and progression of frailty are influenced by various factors, including smoking [17, 18, 43, 44]. Our mouse model of smoking-induced aging, combined with population-based validation studies, highlights the association between smoking, elevated inflammatory markers, and a higher prevalence and incidence of frailty. Evidence suggests that frailty is linked to inflammatory factors, mitochondrial and apoptosis-related proteins (e.g., fibronectin type III domain-containing protein 5 [FNDC5]), as well as neural-related molecules such as granulin precursors [45–48]. In frail individuals, inflammatory markers—including IL-6, TNF-α, CXCL10, and CX3CL1—are often abnormally expressed [45, 49].

This study focused on changes in the expression of inflammation-associated molecules, specifically TNF-α, IL-1β, IL-6, and IL-18, before and after the onset of frailty in a mouse smoking model. Analysis of a smoking-related gene database revealed significant alterations in KEGG signaling pathways, particularly the TNF signaling pathway, underscoring its strong association with inflammation.

We investigated whether TNF-α and IL-1β could serve as candidate molecular markers for characterizing the inflammatory response to smoking and disability. This was achieved by constructing a smoking-induced disability animal model and analyzing transcriptome data from both smokers and nonsmokers. Our findings revealed an upregulation of TNF-α and IL-1β expression in both mouse and human data following cigarette smoke exposure. In contrast, the expression of IL-6 and IL-18 remained relatively unchanged. This disparity may be linked to the activation of pathways, such as NF-κB, by nicotine and other cigarette constituents. These pathways predominantly stimulate the production of proinflammatory cytokines TNF-α and IL-1β, which further amplify NF-κB pathway activity. However, the impact of these pathways on IL-6 and IL-18 appears more nuanced and context-dependent, varying by cell type and stimulatory conditions. In the context of smoking-induced sterile inflammation, the immune response may be subdued, resulting in minimal IL-6 and IL-18 release. These findings suggest that TNF-α, IL-1β, and the NF-κB pathway are critical mediators linking smoking-induced inflammation to frailty. A recent systematic review and meta-analysis on geriatric diseases also supports this, highlighting elevated TNF-α and IL-1β levels as being associated with sarcopenia and frailty [36]. This discovery may pave the way for developing novel biomarkers for senile frailty and advancing therapeutic interventions. Our results further demonstrated that high-dose smoking in mice (16 cigarettes/day, 176 mg/day) was associated with a heightened risk of frailty and inflammation. These findings imply that older individuals who smoke 12 cigarettes/day over a 30-year period are at an elevated risk of frailty. Interestingly, no significant trends were observed in low-dose (8 cigarettes/day, 88 mg/day) or medium-dose (12 cigarettes/day, 144 mg/day) smoking mice. This suggests that a smoking dose threshold must be met to establish a clear correlation between smoking and inflammation.

When smoking exceeded medium-to-high doses (144 mg/day), a correlation between smoking and the inflammatory response became evident. This is likely due to the individualized nature of smoking in humans, which contrasts with the difficulty of fully controlling passive smoking in mice during the construction of an animal model. Notably, future studies will incorporate a whole-body inhalation system to precisely regulate the smoke inhalation time and tar content consumed by the mice, ensuring accuracy while maintaining consistency in total smoking duration and inhalation levels. This adjustment aims to better simulate the autonomous smoking behaviors of humans [50].

**Figure S2.**
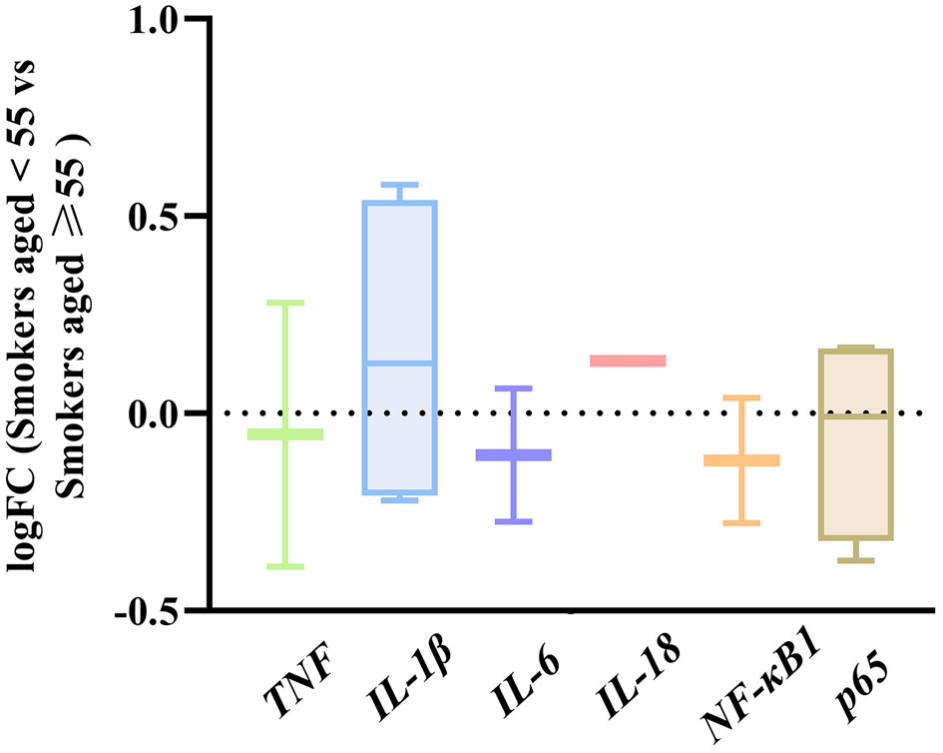
**There is no significant correlation between the age of smokers and the gene expression of inflammatory factors in their lymphocytes.** Smokers from the datasets GSE12585 and GSE18723 on the GEO Accession viewer (nih.gov) were categorized into two age groups: Smokers aged < 55 and smokers aged ≥ 55. Then, the gene expressions of TNF, IL-1β, IL-6, IL-18, NF-κB1, and p65 in their lymphocytes were analyzed. However, no significant difference was observed in the expression levels of these six inflammatory factors (|logFC| ≥ 1.0 considered significant). IL-1β: Interleukin-1β.

**Figure S3.**
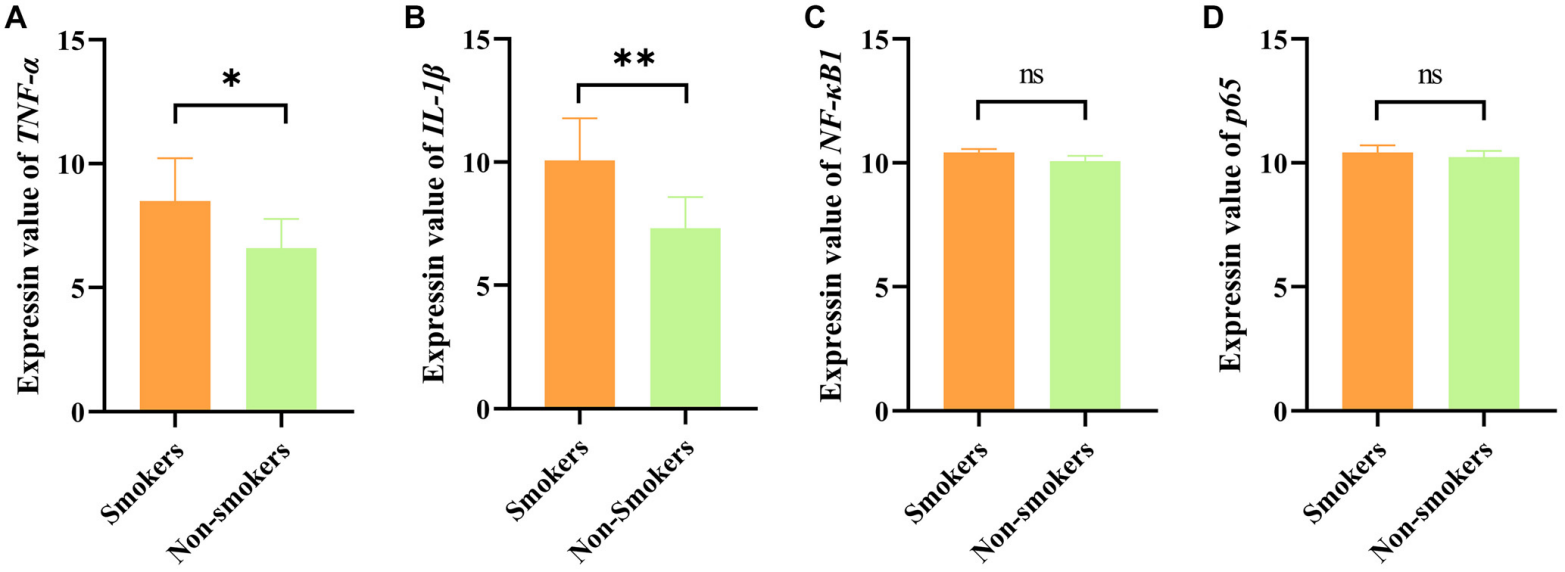
**Gene expression levels of *TNF-α*, *IL-1β*, *NF-κB1*, and *p65* in PBMCs from younger populations.** (A–D) Samples from individuals aged under 55 years were selected from the GSE12585 dataset to analyze the expression of *TNF-α*, *IL-1β*, *NF-κB1*, and *p65* in PBMCs of smokers vs nonsmokers. Significant differences were observed in the expression of *TNF-α* and *IL-1β*, whereas no significant differences were found in the expression of *NF-κB1* and *p65* between the two groups. All data were shown as mean ± SD. *P < 0.05; **P < 0.01; ***P < 0.0005; ****P < 0.0001. TNF-α: Tumor necrosis factor-α; IL-1β: Interleukin-1β; PBMC: Peripheral blood mononuclear cell.

Additionally, we explored the relationship between smoking, aging, and frailty. While no significant differences were observed in the expression of inflammation-related genes within lymphocytes derived from younger vs older smokers (Figure S2), further studies focusing on younger animals are necessary to isolate the effects of smoking from those of aging. To investigate dose-response effects in younger smokers, we analyzed the GSE12585 dataset, identifying subjects under 55 years old and dividing them into smoking and nonsmoking groups. Expression levels of TNF-α, IL-1β, NF-κB1, and p65 were then compared between these groups. Interestingly, TNF-α and IL-1β were significantly elevated in the young smoking group, while no notable changes in NF-κB1 or p65 levels were observed (Figure S3). This divergence may result from interactions with alternative signaling pathways, which differ from those observed in older individuals. Overall, smoking appears to predominantly influence the expression of TNF-α and IL-1β in young smokers, potentially reflecting physiological differences and variations in smoking behaviors within this demographic.

Moreover, alterations in body weight, mouse food intake, lung weight, muscle strength, gait speed, and expression of inflammatory factors can be used to more precisely evaluate frailty in mice. However, these detection methods still possess certain limitations. (1) In our study, during the initial stage of smoke exposure in mice, the lung weight of mice temporarily increased due to inflammatory responses and edema in lungs. Although it is impossible to measure lung weight in humans, lung mass can serve as an indicator in mouse models. However, as the duration of smoke exposure increases and pathological changes intensify, mouse lung tissue may undergo atrophy and fibrosis, leading to a gradual decrease in lung mass. So far, lung mass as an indicator may not be suitable for longer-term smoke exposure experiments. (2) In humans, lung function deterioration indicators are directly measured to verify smoking influence on lung tissues, such as increased airway resistance, lung infections, and even lung cancer. These lung function indicators do not be detected based on lung weight, and thus more precise assays, including mouse airway resistance and lung compliance analysis systems, will be applied in subsequent studies. (3) Measurements, such as muscle strength and gait speed do not adequately reflect changes in cognitive function in mice. Thus, we would incorporate additional indicators, such as the open field test, T-maze or Y-maze tests, and the Morris water maze test, to further assess the activity levels and cognitive function of smoking mice, thereby much precisely reflecting frailty in mice [51]. (4) Detection indicators such as food intake are significantly influenced by individual differences among mice, leading to larger errors when the sample size is small. It is preferable to use such indicators when the sample size is larger. (5) The current smoke exposure model possesses certain limitations. The intake of cigarette smoke components varies due to individual differences among mice. So far, in addition to measuring behavioral and inflammatory indicators in mice, the degree of smoking exposure using serum cotinine levels should be quantified [29] to well monitor the correlation between smoking and frailty in the elderly. In general, it is worthwhile to further investigate how to better control the smoking behavior of mice in a smoking model and improve the construction of a smoking-induced disability model.

In our study, old aged mice exhibited a tendency toward frailty even in the absence of cigarette smoke exposure. We identified two primary factors that could have significant impacts on this observation. (1) The advanced age of the mice may have initiated spontaneous frailty. (2) During the experimental procedure, the handling and relocation of mouse cages by experimenters may elicit a certain degree of stress response in the mice. Over an extended experimental duration, this stress may exert an influence on the physiological state of the mice, leading to a decrease in indicators such as grip strength among the elderly mice within the control group.

### Limitations and future perspectives

The current study lacks validation experiments examining the suppression of TNF-α and IL-1β expression in relation to frailty changes, as well as their effects on the NF-κB inflammatory signaling pathway in smoking mice. To address this, future research will investigate whether this phenomenon is also present in younger smoking populations.

## Conclusion

This study demonstrates that long-term smoking, especially at high doses, leads to increased levels of TNF-α and IL-1β in aged mice and humans. These elevated levels subsequently promote the activation of NF-κB inflammatory signaling and contribute to the development of frailty in the elderly. In high-dose smoking mice, the decline in grip strength was 2.62 times greater than in nonsmoking mice, while the reduction in drop time was 2.94 times greater. Elevated serum levels of TNF-α and IL-1β can serve as quantitative indicators for the onset and progression of smoking-induced frailty.

## Supplemental data

## Data Availability

Raw data supporting the conclusions of this study will be made available by the authors upon reasonable request.
